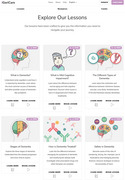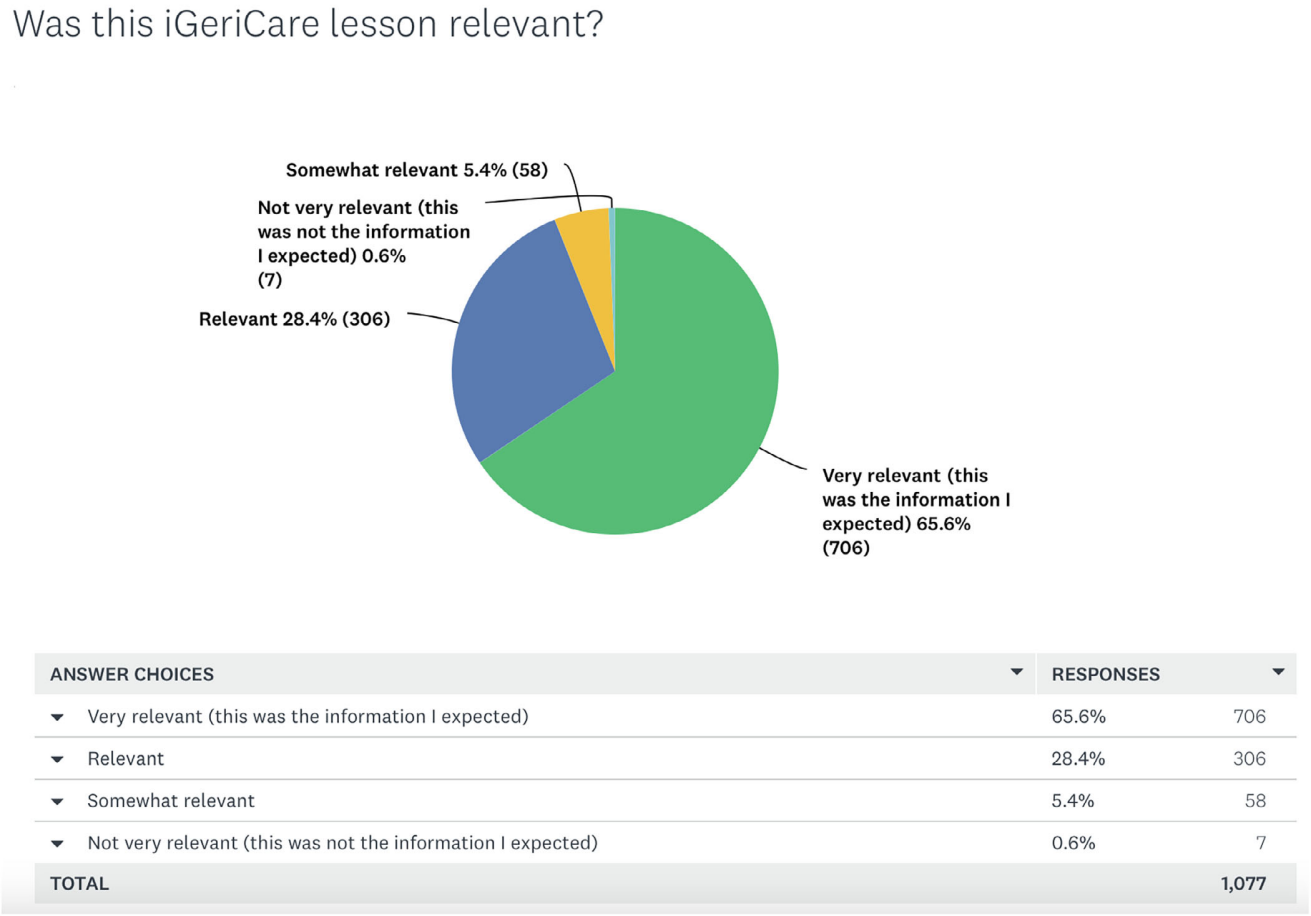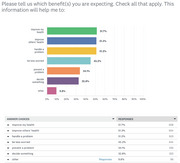# Impact and Perceived Value of iGeriCare e‐Learning Among Dementia Care Partners and Others: Evaluation Using the IAM4all Questionnaire

**DOI:** 10.1002/alz70858_103263

**Published:** 2025-12-24

**Authors:** Anthony J. Levinson, Stephanie Ayers, Sandra Clark, Rebekah Woodburn, Roland Grad, Alexandra Papaioannou, Richard Sztramko

**Affiliations:** ^1^ McMaster University, Hamilton, ON, Canada; ^2^ McGill University, Montreal, QC, Canada

## Abstract

**Background:**

Dementia is a growing global health issue; informal care partners provide the majority of care for individuals living with dementia, often at significant personal, emotional, and financial cost. Care partners often lack access to appropriate educational resources and support systems. Web‐based educational interventions have the potential to support care partners, but existing programs are often limited by scope, language, or accessibility.

**Method:**

iGeriCare.ca, an evidence‐based, freely available, internet‐based program designed to educate dementia care partners at their own pace, was developed by experts from McMaster University. iGeriCare features 12 multimedia lessons, email‐based micro‐learning, and live events with expert interaction. Uptake and engagement are measured using web and learning analytics, and the IAM4all questionnaire is used to assess web‐based health information outcomes.

**Result:**

Since July 2018, iGeriCare has over 227,000 unique users, more than 337,800 sessions, and 615,000 page views. The lessons have been accessed over 58,600 times. The 52‐week email‐based micro‐learning series has had more than 3,050 subscribers. Thirty‐eight live events have been hosted and recordings have been watched > 52,600 times. Since March 2021, 1,077 IAM4all responses have been received from care partners (38%), those with dementia or concerned about their cognitive health (29%), and health care providers/trainees (24%). 94% found the lessons relevant, and 99% reported understanding the content well. 61% reported learning something new, and 58% felt motivated to learn more. Respondents also reported feeling validated (50%), reassured (46%), or said the content refreshed their memory (37%). 98% reported intention to use a lesson, including: to better understand something (73%), discuss the information with someone else (53%), or do things differently (38%). 95% expected to benefit from the information, including to improve their health (52%), another person's health (51%), or handle a problem (51%).

**Conclusion:**

iGeriCare represents an innovative and scalable solution to meet the educational needs of care partners. By leveraging evidence‐based instructional design, iGeriCare empowers care partners with the knowledge and confidence to manage their responsibilities effectively. Future analyses of a recently completed pilot randomized controlled trial will further explore its impact on care partner outcomes.